# OLIDIAG Study: Extra Virgin Olive Oil Supplementation in the Diet of Women with Gestational Diabetes Mellitus—A Randomized Clinical Trial

**DOI:** 10.3390/nu18071120

**Published:** 2026-03-31

**Authors:** Alicia Jawerbaum, Silvia Gorban de Lapertosa, Magdalena Rey, Inés Argerich, Mariano Reynoso, María Celeste Muntaner, Celina Bertona, Verónica Kojdamanian Favetto, Esteban Díaz, Stella Sucani, Dalmiro Gomez Ribot

**Affiliations:** 1 Laboratory of Reproduction and Metabolism, Centro de Estudios Farmacológicos y Botánicos (CEFYBO), Consejo Nacional de Investigaciones Científicas y Técnicas (CONICET), Facultad de Medicina, Universidad de Buenos Aires (UBA), Ciudad Autónoma de Buenos Aires 1121, Argentina; dalmiro.g.ribot@gmail.com; 2 Centro de Investigaciones Farmacológicas, Facultad de Medicina, Universidad Nacional del Nordeste, Corrientes 3400, Argentina; dralapertosa@hotmail.com; 3 Hospital Alemán, Ciudad Autónoma de Buenos Aires 1118, Argentina; mrey@hospitalaleman.com; 4 Hospital Perrupato, Mendoza 5570, Argentina; maria.argerich@um.edu.ar; 5 Cátedra de Biología Celular y Molecular, Departamento de Ciencias Biológicas, Facultad de Farmacia y Bioquímica, Universidad de Buenos Aires (UBA), Ciudad Autónoma de Buenos Aires 1113, Argentina; mreynoso@docente.ffyb.uba.ar; 6 Unidad Patagónica de Medicina Materno Fetal y Embarazo de Alto Riesgo, Neuquén 8300, Argentina; maria.muntaner@facimed.uncoma.edu.ar; 7 Hospital Universitario, Universidad Nacional de Cuyo (UNCUYO), Mendoza 5502, Argentina; celina.bertona@hospital.uncu.edu.ar; 8 Center for Education, Prevention and Care for Patients with Diabetes (CEPA), Municipalidad de Pilar, Pilar 1629, Buenos Aires, Argentina; veronica.kojdamanian@hospitalitaliano.org.ar; 9 Hospital General de Agudos Dr. Ignacio Pirovano, Ciudad Autónoma de Buenos Aires 1428, Argentina; estebandiaz_17@hotmail.com; 10 Hospital Materno Provincial Dr. Felipe Lucini, Córdoba 5006, Argentina; sucanism1@hotmail.com

**Keywords:** gestational diabetes mellitus, extra virgin olive oil, dietary intervention, insulin requirement, triglyceridemia, maternal–neonatal outcomes, perinatal complications

## Abstract

Background/Objectives: Gestational diabetes mellitus (GDM) is a prevalent metabolic disease associated with maternal and neonatal complications. Diets enriched with extra virgin olive oil may benefit metabolism and provide antioxidant effects. We aimed to evaluate the effects of dietary supplementation with extra virgin olive oil on metabolic parameters and insulinization rate in women with GDM. Methods: This is a multicenter, parallel, randomized controlled trial in which 190 patients with GDM were enrolled before week 29 of gestation and randomized into the Control group and the Intervention group. Patients in the Intervention group received the indication to consume three tablespoons of extra virgin olive oil (EVOO) daily. At term, metabolic parameters, insulin requirement and maternal and neonatal outcomes were evaluated. Results: Control and Intervention groups showed no differences in maternal age (31.7 ± 6.0 and 32.4 ± 5.2 years, respectively) or gestational age (26.5 ± 3.6 and 26.7 ± 3.3 weeks, respectively) at enrollment. Primary outcomes showed that EVOO consumption was associated with a reduction in insulin requirement (RR 0.595, 95% CI 0.361–0.967, *p* < 0.05). There was a significant reduction in triglyceridemia in the EVOO-supplemented group compared to controls (MD −43.3 mg/dL, 95% CI −66.8–−19.8, *p* < 0.01). There were no effects of the intervention on gestational weight gain. As secondary outcomes, maternal BMI and gestational age at delivery showed no changes between the groups. Although maternal and neonatal composite outcomes were not significantly reduced, the rate of neonates with more than one complication (RR 0.340, 95% CI 0.133–0.870, *p* < 0.05) and NICU requirement (RR 0.367, 95% CI 0.140–0.939, *p* < 0.05) were significantly reduced in the Intervention group. Conclusions: In GDM, maternal dietary supplementation with extra virgin olive oil resulted in reduction in maternal triglyceridemia, need of insulinization and neonatal complications.

## 1. Introduction

Gestational diabetes mellitus (GDM), defined as carbohydrate intolerance resulting in hyperglycemia first diagnosed during pregnancy, is a common pregnancy complication that affects 17% of pregnancies worldwide [1]. Key risk factors include maternal obesity, prior GDM, a family history of type 2 diabetes (DM2), polycystic ovary syndrome and ethnicity [2]. GDM is associated with adverse outcomes for both mother and child. In the short term, it increases the risk of several adverse maternal outcomes such as excessive weight gain, cesarean birth and preeclampsia, as well as adverse neonatal outcomes including macrosomia, birth injuries, hypoglycemia and respiratory distress syndrome [2]. In the long term, GDM predisposes the mother and her offspring to obesity, DM2 and cardiovascular disease [2,3].

Although the etiology of these adverse outcomes is complex and multifactorial, the maternal hyperglycemic state has been clearly related to an increased prooxidant and proinflammatory environment involved in the induction of maternal, fetal and offspring complications [4,5]. Extra virgin olive oil (EVOO) is a relevant functional food with nutrigenomic properties [6]. Out of pregnancy, EVOO supplementation has been shown to prevent cardiovascular diseases and other pathologies related to the prooxidant and proinflammatory environment [7,8]. EVOO is a main component of the Mediterranean diet, a dietary pattern considered beneficial to health both out of pregnancy and during pregnancy [9,10]. In pregnancy, studies have evidenced the capacity of a diet enriched in EVOO and pistachios to reduce the incidence of GDM, whereas offspring of these studies showed improved health at six years of age [11,12]. Previously, we performed preclinical studies in experimental models of diabetes and pregnancy and identified antioxidant and anti-inflammatory effects of EVOO-enriched diets in placentas, fetuses and offspring of diabetic rats [13,14,15]. In addition, our translational studies performed in women with GDM showed that EVOO addition to the maternal diet led to a reduction in the placental proinflammatory state and to a reduction in maternal hypertriglyceridemia in a cohort of women with GDM [16,17].

Building on the results of previous investigations and considering that dietary intervention remains a cornerstone of GDM management, the aim of the present study was to conduct a national multicenter randomized clinical trial to test the hypothesis that a maternal diet enriched in EVOO reduces maternal insulinization, triglyceridemia and maternal weight gain in GDM mothers. Secondarily, we aimed to explore the effects of EVOO-enriched diets on maternal and neonatal outcomes in the women with GDM.

## 2. Materials and Methods

### 2.1. Study Population and Design

The OLIDIAG Clinical Trial is an interventional randomized multicentric study trial that was performed in eight centers located in five Argentinian provinces. This trial was first approved by the Ethics Committee of the School of Medicine, National Northeast University on 15 December 2020 (Project Review Board 10-2020-1941 Resolution 58/20) and was prospectively registered within clinicaltrials.gov (NCT05120388). The study was conducted in accordance with the ethical principles of the Declaration of Helsinki (1964, and subsequent amendments up to 2024 [18]) and adhere to the CONSORT guidelines (Supplementary material: CONSORT 2025 checklist). Participants were enrolled from November 2021 to April 2024.

**Figure 1:**
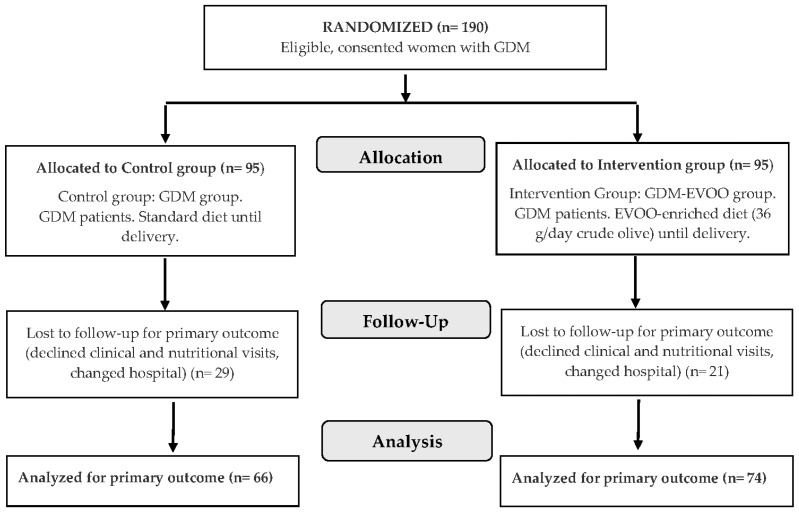
Flow chart of the OLIDIAG study. Abbreviations: GDM: gestational diabetes mellitus, EVOO: extra virgin olive oil.

Pregnant adult women with GDM were enrolled before 29 weeks of pregnancy. GDM was diagnosed according to the Latin American Association of Diabetes (ALAD) and the Argentine Society of Diabetes (SAD) criteria, by fasting glycemia values 100–126 mg/dL in two measurements, or after a universal 75 g oral glucose tolerance test (≥140 mg/dL at 2 h) [19]. Exclusion criteria included multiple pregnancy, preexisting bariatric surgery, preexisting diseases including type 1 or type 2 pregestational diabetes and infectious diseases. At each of the eight participating centers, the protocol was explained to women with GDM, who were invited to participate in the study and signed the consent form for further eligibility evaluation. As summarized in Figure 1, a total of 190 eligible women with GDM accepted to participate in the protocol and signed the consent form. At inclusion, women in each center were randomly allocated to either the standard care Control group (*n* = 95) or the Intervention group (*n* = 95). Randomization was conducted in random blocks of 4/6, computed generated, with allocation 1:1 to the Intervention or Control group at each of the eight trial centers.

Women in both arms received standardized personal clinical and nutritional care in accordance with the national and international guidelines [19,20]. All participants received nutritional and lifestyle counseling, instructions for glucose monitoring, and insulin as medication, if required.

### 2.2. Nutritional Intervention

Women in the EVOO-enriched group were instructed to consume three 13 mL tablespoons of extra virgin olive oil daily (36 g/day). The instructions to consume the EVOO uncooked and within the main meals were provided. The study was not blinded, as those women in the EVOO-enriched group were provided with commercial EVOO bottles at the nutrition visits to ensure adherence. Women in the Control group were not instructed to consume EVOO and did not include EVOO in their diets due to cultural and economic reasons. In both the Control and Intervention groups, follow-up appointments with the obstetrics and nutrition professionals in charge of each center were monthly or more frequent according to the gestational age and requirements. An objective outcome assessment and standardized data collection were performed, with professional evaluation of nutritional adequacy against recommended energy and macronutrient intakes during pregnancy [19,20,21]. The clinical data of the participants was recorded at each obstetric visit. Adherence to the EVOO dietary intervention and intake of calories and macronutrients were evaluated at each nutritional visit. Adherence was considered good if EVOO daily consumption was over 26 g/day 5 to 7 times a week, regular if EVOO daily consumption was over 26 g/day 3 or 4 times a week and bad if EVOO daily consumption was lower than 26 g/day or 2 times a week or lower. Clinical data obtained on the third trimester of pregnancy (at the last obstetric and nutritional visits) and at delivery were evaluated.

### 2.3. Outcomes

The primary endpoints were maternal insulinization rate, maternal triglyceridemia and gestational weight gain. The secondary outcomes included maternal BMI, gestational age at delivery, triglycerides to HDL cholesterol ratio and metabolic control, as well as maternal and neonatal outcomes identified as critically important in the care of pregnant women [22,23]. Maternal outcomes were evaluated as a composite (hypertensive complications, placental complications, cholestasis, hemorrhages, and preterm birth) and individually considered. Cesarean section rate was also evaluated. Neonatal outcomes were evaluated as a composite (hypoglycemia, hyperbilirubinemia, respiratory distress syndrome, shoulder dystocia, perinatal death and neonatal care intensive unit (NCIU) requirement), with one, more than one, and individual complications considered. Large for gestational age (above the 90th percentile for gestational age), small for gestational age (below the 10th percentile for gestational age) and neonatal weight were also evaluated.

### 2.4. Statistical Analysis

An a priori sample size calculation was performed considering the primary outcomes of the trial, the expected difference between groups and the variability reported in preliminary studies [16]. The larger of the sample size requirements was adopted. To account for an anticipated dropout rate of 30%, the total recruitment target was set at 190 participants. This target was set considering a two-sided significance level of α = 0.05, 80% power, and a 1:1 allocation ratio.

Data are presented as percentages for categorical variables and mean ± SD for continuous variables. Baseline comparisons: Continuous variables were analyzed using one-way ANOVA. Assumptions of normality and variance homogeneity were evaluated using the Shapiro–Wilk test and the Levene test, respectively. All variables met these assumptions except fructosamine, which violated variance homogeneity and was therefore analyzed using Kruskal–Wallis’s test. Categorical variables were evaluated using chi-square or Fisher’s exact tests, as appropriate. Outcomes at term: Continuous outcomes were examined using one-way analysis of covariance (ANCOVA). Both unadjusted and adjusted analyses were performed to improve precision and account for clinically relevant covariates, with randomized group as the main factor and gestational age at delivery and pre-pregnancy BMI as covariates, when applicable. Assumptions were evaluated using the Shapiro–Wilk test (normality), the Levene test (variance homogeneity), and the Group × Covariate interaction test (homogeneity of regression slopes). Adjusted means were compared using the Bonferroni’s post hoc test. Mean differences (MDs) between groups with 95% confidence intervals (95% CI) were calculated for continuous outcomes. For categorical outcomes at term, preliminary logistic regression analyses were performed to assess the potential need for covariate adjustment. Covariates did not contribute significantly to these models, and adjusted estimates did not differ meaningfully from unadjusted ones. Consequently, chi-square or Fisher’s exact tests were used for the final analysis. Relative risks (RRs) and 95% confidence intervals (95% CI) were calculated for dichotomous outcomes.

Time to insulin initiation was analyzed using Kaplan–Meier survival curves and compared between groups using the log-rank test. Hazard ratios (HRs) with 95% confidence intervals (96% CI) were estimated using Cox proportional hazards regression models, both unadjusted and adjusted for pre-pregnancy BMI.

A strict intention-to-treat analysis was not applied, as no imputation processes were applied considering the patients that dropped out in the analysis. These patients declined nutritional and clinical visits and changed hospitals, with no data obtained on their third trimester of pregnancy. Analysis was performed in all patients with third trimester data, regardless of their adherence to the EVOO supplementation diet. Outliers, identified by the Grubbs method, were less than 5%.

All statistical analyses were performed using InfoStat software (version 2017, Grupo InfoStat, Facultad de Ciencias Agropecuarias, Universidad Nacional de Córdoba, Argentina), GraphPad Prism (version 8.0, GraphPad Software, San Diego, CA, USA) and R statistical software through RStudio (RStudio 2026.01.1+403 “Apple Blossom” Release; Posit Software, Boston, MA, USA). A *p* value lower than 0.05 was considered statistically significant.

## 3. Results

### 3.1. Study Population

As shown in the study Flow chart (Figure 1), 190 women with GDM were randomized into the Control and Intervention groups, but 30% (29 patients) and 22% (21 patients), respectively, discontinued the study due to missed clinical and nutritional visits and changed hospitals, resulting in no data obtained on the third trimester of pregnancy. All patients in the Control and Intervention groups with data on the third trimester of pregnancy were evaluated in this study (140 patients): 66 patients were analyzed in the GDM group (Control) and 74 in the GDM-EVOO group (Intervention). Nutritional evaluation of adherence to the diet showed 82.4% of good adherence, 9.5% of regular adherence and 8.1% of bad adherence in the Intervention group. All randomized participants in the Intervention group with term data were analyzed despite the degree of adherence to the EVOO-enriched diet. In this multicenter study, we found no significant differences between the centers for the parameters evaluated.

### 3.2. Clinical and Biochemical Baseline Data

The baseline characteristics of the OLIDIAG participants are reported in Table 1. At enrollment, there were no differences in maternal age, pre-pregnancy BMI, the proportion of smokers, or the proportion of obesity (BMI > 30 kg/m^2^) between the Control the Intervention groups. Proportion of nulliparity and the gestational age at enrollment were also similar between the groups (Table 1). Regarding metabolic parameters at enrollment, fasting glucose levels, HbA1c levels, fructosamine, and triglyceridemia were similar in the GDM patients who received or did not receive the EVOO-supplemented diet (Table 1).

### 3.3. Primary Outcomes

The effects of dietary supplementation with EVOO on the primary outcomes of the OLIDIAG study are shown in Table 2. Some patients received clinical indications to initiate insulinization on the first clinic visit, together with the indication of EVOO treatment and thus, prior to the initiation of the EVOO consumption. No significant changes in the rate of insulinization at first clinic visit were observed in the GDM patients of the Control group compared to the Intervention group (Table 2). Insulin requirement at pre-delivery was first evaluated including the patients who initiated insulin treatment at their first clinical visit, when EVOO supplementation was indicated but not yet initiated. The insulin requirement was not significantly reduced in the Intervention group (41.9%) compared to the Control group (54.6%) (RR 0.758, 95% CI 0.534–1.085, *p* = 0.135) when patients who initiated insulin treatment at their first clinical visit and thus did not previously initiate EVOO consumption were included (Table 2).

Interestingly, when the insulin requirements were evaluated after the patients initiated the EVOO treatment, and thus during the intervention, a significant reduction in the need for insulin in the Intervention group compared to controls was observed (GDM group: 40.9%, GDM-EVOO group: 24.3%, 95% CI 0.361–0.967, *p* = 0.036) (Table 2). Relative risk analysis showed a 40% reduced risk of insulinization after the mother initiates the treatment with the EVOO-supplemented diet.

Time-to-event analysis was performed to evaluate the time from initiation of EVOO supplementation to initiation of insulin therapy. Kaplan–Meier curves showed that the Intervention group remained free of insulin therapy for a significantly longer period compared with the Control group, with a cumulative probability of remaining free of insulin therapy declining earlier and more markedly in the Control group (*p* = 0.005). The median time to insulin initiation was 6.5 weeks in the Control group, while the median was not reached in the Intervention group as more than half of the participants in this group remained free of insulin therapy until delivery (Table 2). To further evaluate the effect of the intervention on timing of insulin initiation, Cox proportional hazards regression models were performed, revealing a significantly lower hazard of insulin initiation in the Intervention group compared with the Control group (HR 0.227, 95% CI 0.074–0.698, *p* = 0.010). This effect remained significant after adjustment for pre-pregnancy BMI (Table 2). No differences in insulin dosage were observed between the groups (Table 2).

There was a significant reduction in triglyceridemia in the Intervention group compared to the Control group (MD −43.3 mg/dL, 95% CI −66.8–−19.8, *p* = 0.002), a reduction that remained significant when values were adjusted for gestational age at delivery and pre-pregnancy BMI (MD −43.0 mg/dL, 95% CI −68.6–−17.4, *p* = 0.005) (Table 2).

There were no effects of the intervention on gestational weight gain when compared to the Control group, both considering unadjusted values and values adjusted by gestational age at delivery and pre-pregnancy BMI (Table 2).

### 3.4. Maternal Outcomes

As secondary outcomes, maternal clinical and metabolic parameters were evaluated at term in the OLIDIAG patients. As shown in Table 3, term maternal BMI and gestational age were similar in the Control and the Intervention groups when both unadjusted values and values adjusted for gestational age at delivery or pre-pregnancy BMI were considered.

Regarding metabolic control, good mean metabolic control was similarly observed in the GDM patients who received or did not receive the EVOO supplementation, as shown by fasting glucose, fructosamine and HbA1c values (Table 3).

There was a significant reduction in the triglyceride/HDL cholesterol ratio in the Intervention group compared to the Control group (MD −0.93, 95% CI −1.46–−0.40, *p* = 0.001), a reduction that remained significant when values were adjusted for gestational age at delivery and pre-pregnancy BMI (MD −1.03, 95% CI −1.65–−0.41, *p* = 0.002) (Table 3).

As changes in metabolic parameters may reflect changes in intake of calories and macronutrients, the rate of excessive caloric intake (≥110% of recommended energy intake), insufficient caloric intake (≤90% of recommended energy intake), excessive carbohydrate intake (≥65% of recommended energy intake), insufficient protein intake (≤10% of recommended energy intake), excessive total fat intake (≥35% of recommended energy intake), excessive saturated fat intake (≥10% of recommended energy intake) and excessive polyunsaturated fatty acids (PUFAs) intake (≥11% of recommended energy intake) were evaluated, showing no changes in the GDM patients who received or did not receive the EVOO supplementation (Table 3).

Regarding maternal complications, the evaluation of a maternal composite including hypertensive complications, placental complications, cholestasis, hemorrhages and preterm birth showed no significant changes between the patients in the GDM group (25.8%) and the GDM-EVOO group (17.6%) (RR 0.682, 95% CI 0.363–1.280, *p* = 0.238) (Table 3), with no significant reductions in the individual complications of this composite observed in the Intervention group (Table 3).

The evaluation of delivery mode showed a high rate of cesarean section in the evaluated population (GDM group: 67.7%, GDM EVOO group 58.1%), with no significant changes between the groups (RR 0.872, 95% IC 0.669–1.132, *p* = 0.383). Rates for non-elective cesarean section and patients with labor were also non-significantly different (Table 3).

### 3.5. Neonatal Outcomes

As secondary outcomes, neonatal parameters were evaluated in the OLIDIAG Control and Intervention groups. As shown in Table 4, there were no differences in the rate of neonates large for gestational age and small for gestational age. Main neonatal weight was unchanged in both groups when unadjusted values were considered (MD −102 g, 95% CI −255–51, *p* = 0.194), but it was significantly reduced in the Intervention group when adjusted for gestational age at delivery and pre-pregnancy BMI (MD −147 g, 95% CI −291–−3, *p* = 0.047).

Regarding the neonatal complications, a composite including hypoglycemia, hyperbilirubinemia, respiratory distress syndrome, shoulder dystocia, stillbirth and neonatal intensive care unit (NICU) requirements were evaluated (Table 4). Although the evaluation of the neonatal composite showed no significant changes between the patients of the GDM group (25.7%) and the GDM-EVOO group (18.9%) (RR 0.730, 95% CI 0.396–1.359, *p* = 0.331), when neonates with more than one complication were considered, there was a significant reduction in the EVOO-supplemented GDM group compared to the Control group (GDM group: 19.7%; GDM-EVOO group: 6.8%, 95% CI 0.133–0.870, *p* = 0.041). Relative risk analysis showed a 66% reduced rate of neonates with more than one complication in the GDM group supplemented with EVOO compared to controls (Table 4).

Neonatal complications were then considered individually. There were no cases of shoulder dystocia. There was one case of stillbirth observed in the Control group. No significant differences in the cases of hypoglycemia, hyperbilirubinemia and respiratory distress syndrome were observed between the groups. (Table 4).

Regarding NICU requirement, it was significantly reduced in the Intervention group compared to controls (GDM group:18.2%, GDM-EVOO group: 6.8%, 95% CI 0.140–0.939, *p* = 0.036). The relative risk analysis showed a 63% reduced risk of NICU requirement when the GDM mothers received the EVOO supplementation (Table 4).

This important reduction in NICU requirement led us to further address the sub-population of neonates with complications (*n* = 30). In this population, NICU requirement was due to respiratory distress syndrome, hyperbilirubinemia or neonatal hypoglycemia (Table 4).

Considering the neonates with these complications (*n* = 30), there was a significant reduction in NICU requirement in the GDM-EVOO group (35%) compared to controls (75%) (95% CI 0.210–0.935, *p* = 0.03), showing the relative risk analysis a 52% reduced risk of NICU requirement in the neonates with complications in the EVOO-supplemented group (Table 4). When the population of neonates with respiratory distress syndrome was considered (*n* = 9), NICU rates showed no significant changes (GDM: 100%, GDM-EVOO: 67%) (Table 4). When the population of neonates with hyperbilirubinemia was considered (*n* = 16), NICU rates were similar in both groups (GDM: 57.1%, GDM-EVOO: 55.6%). When the population of neonates with hypoglycemia was considered (*n* = 12), NICU rates were significantly reduced (GDM: 100%, GDM-EVOO: 33%, 95% CI 0.097–0.760, *p* = 0.014), showing the relative risk analysis a 66.7% reduced risk of NICU requirement in the neonates with hypoglycemia (Table 4).

## 4. Discussion

As primary results, the OLIDIAG study demonstrated that a dietary supplementation with extra virgin olive oil in GDM pregnancies reduced maternal triglyceridemia and the need for insulinization. Furthermore, secondarily, we found that the benefits of the EVOO-enriched diet reach the developing fetus, as evidenced by the lower rate of neonates requiring NICU observed in the Intervention group.

In this study, baseline characteristics were similar in the Control and Intervention groups. Obesity was not excluded from the study and showed similar rates (44–40%) to those reported at reproductive age in other national studies and in diverse populations [24,25]. Maternal nutrition is crucial for metabolic control in GDM, and lifestyle education and follow-up of nutritional advice have largely improved the metabolic control in this prevalent gestational disease [26,27]. The fact that the women in this study should attend monthly visits to clinicians and nutritionists is probably related to the good metabolic control observed in both the Control and the Intervention groups.

To our knowledge, this is the first study to address the effects of dietary supplementation with EVOO on maternal and neonatal outcomes in patients with GDM. Prior to this multicenter study, we conducted a preliminary study in which an EVOO-enriched diet was indicated to a cohort of GDM patients for further evaluation of the placentas, showing potent anti-inflammatory effects and regulation of the nuclear receptors PPARs in the placentas of patients with GDM that received the EVOO-enriched diet [16,17]. EVOO is mostly composed of oleic acid (70%), a monounsaturated fatty acid that activates the nuclear receptors PPARs, leading to metabolic, antioxidant and anti-inflammatory effects [4,28]. In addition, EVOO is rich in polyphenols with potent antioxidant effects [6]. Outside of pregnancy, the nutrigenomic and epigenetic effects of EVOO have been reported extensively, as have the reported clinical benefits of EVOO in cardiovascular and proinflammatory diseases [6,7,8,29]. In pregnancy, a metanalysis study showed that adherence to the Mediterranean diet style, rich in EVOO, in high-risk pregnancies leads to a reduction in the induction of GDM [10].

The present study evaluated pregnant women with GDM, and, as primary results, showed that an EVOO-supplemented diet reduced hypertriglyceridemia, a result that was significant even when adjusted for pre-pregnancy BMI. Reduction in hypertriglyceridemia has been reported in the Mediterranean diet and EVOO-enriched diets in non-pregnant individuals with different diseases [30,31,32]. In pregnancy, increased triglycerides are related to an increased risk of GDM, preeclampsia, preterm birth and large for gestational age [33,34,35,36], and thus, the observed capability of EVOO supplementation to reduce maternal triglyceridemia is considered relevant.

On the other hand, although insulin is key to maternal metabolic control in GDM, several issues are involved in its usage, including costs, maternal risks of hypoglycemia, the need for intensified obstetric and endocrinologic surveillance, and the emotional burden experienced by women with GDM requiring insulin therapy [37,38]. Therefore, as another primary result of this work, the reduction in the need for maternal insulinization to achieve metabolic control in the patients who consumed the EVOO-enriched diet is considered important. Of note, these benefits were not attenuated when the data were adjusted for pre-pregnancy BMI.

Likely related to the reduced need for insulinization, the triglyceride/HDL cholesterol ratio, a marker of insulin resistance [39], was found reduced in the Intervention group, and these benefits were observed even when gestational weight gain was found unchanged at term in the patients with GDM that received the EVOO-enriched diet compared to controls.

The observed benefits on the evaluated primary outcomes are not likely to be related to changes in energy intake or consumption of other macronutrients, as no changes in the evaluated nutritional parameters were observed between the patients with GDM who received or did not receive the EVOO-enriched diet.

Regarding the evaluated secondary outcomes, no significant reductions in composite or individual maternal outcomes were observed in the present study. Most of the maternal complications reported in the GDM group were similar to those found in other GDM populations, whereas Cesarean section rates are high in Argentina when related to that observed in other countries, possibly due to cultural differences [24,40].

Regarding the neonatal outcomes, no significant changes were observed in the rates of large and small for gestational age between the groups. The main neonatal weight in both groups was normal, and the slight reduction in weight observed in the Intervention group was not associated with increases in neonatal complications, suggesting no complications associated. On the other hand, in this study, the rate of large for gestational age was less than expected for previous studies in GDM patients [24], probably related to the good metabolic control in both groups and the programmed monthly nutritional visits in both arms. Thus, further studies in populations with higher rates of neonates large for gestational age are needed to evaluate whether EVOO-induced reduction in neonatal weight may favor neonates prone to macrosomia.

Interestingly, in this work, although composite neonatal outcomes were not significantly reduced by the maternal EVOO supplementation, both the rate of neonates with more than one complication and the NICU requirement were significantly reduced in the Intervention group, suggesting important benefits for neonatal health, and likely relevant for their long-term life. These results prompted us to address the population of neonates with complications in this study, in which we found that the maternal EVOO-enriched diet led to reduced need for NICU admission in neonates with complications and to a significant reduction in NICU admission rates in neonates with hypoglycemia. However, due to the relatively small absolute number of neonatal complications, these results are preliminary observations that should be further investigated. In addition, further studies are needed to address the long-term effects of this dietary treatment on the offspring.

As potential mechanisms that deserve further study, the reduced insulin-resistant state of the mother may improve in-utero development and neonatal health. Alternatively, direct effects of EVOO components in the maternal diet may lead to beneficial effects in the placenta and the fetuses that impact newborns. In this regard, the Mediterranean diet has been suggested to trigger protective epigenetic mechanisms that could prevent metabolic diseases in offspring [41]. Previously, our preclinical studies in animal models of diabetes and pregnancy demonstrated the benefits of diets enriched in EVOO on the placenta, fetus and offspring [13,14,15], whereas metabolic and anti-inflammatory effects of this diet were previously observed in placentas from GDM patients [16,17].

This work has several limitations. First, the relatively high dropout rate and the lack of a strict intention-to-treat analysis, which could introduce attrition bias. The cause of dropout was a decline in nutritional and clinical visits and changes in hospitals, leading to no data on the third trimester of pregnancy for these patients.

Another limitation is that we were unable to blind the intervention because the Intervention group received bottles of commercial EVOO. This lack of blinding may introduce the possibility of performance and detection bias. Placebos were not provided. In fact, although refined olive oil is depleted of polyphenols, its similar content of monounsaturated fatty acids may lead to partial effects [6]. Other oils may have different effects due to their different fatty acid content and may exceed the recommended content of dietary PUFA in pregnancy if provided in the same amount [20]. Another limitation of the study is the lack of use of biomarker of olive oil consumption.

The possibility that the intervention displaced unhealthy foods is also a limitation. However, as a strength, in this work we observed no changes in excessive or insufficient caloric intake or macronutrients, together with no differences in glucose metabolic control between the groups, suggesting that there were no changes in nutrition and insulinization as a bias due to the lack of blinding and that the changes observed in this study are not likely related to differences in energy intake or consumption of other macronutrients.

## 5. Conclusions

A reduction in maternal insulin requirement and triglyceridemia, as well as fewer newborn complications was evidenced in patients with GDM that received an extra virgin olive oil-supplemented diet. Taken together, our results suggest the potential benefits of EVOO dietary supplementation as a nutritional tool for improving the health of mothers with GDM and their newborns. Therefore, the OLIDIAG study has important clinical implications as a feasible dietary treatment that provides benefits to mothers with GDM and their newborns.

## Figures and Tables

**Table 1:** Baseline characteristics of OLIDIAG participants.

	Control Diet GDM Group (*n* = 66)	Intervention Diet GDM-EVOO Group (*n* = 74)	*p* Value
Maternal age (years)	31.7 ± 6.0	32.4 ± 5.2	0.461
Pre-pregnancy BMI (kg/m^2^)	30.4 ± 6.5	29.8 ± 6.7	0.584
Smokers (%)	11 (13.6)	9 (14.9)	0.836
Obesity (%)	29 (43.9)	30 (40.5)	0.684
Parity (% nulliparity)	16 (24.3)	21 (28.4)	0.579
Gestational age at enrollment (weeks)	26.5 ± 3.6	26.7 ± 3.3	0.732
Fasting glucose at enrollment (mg/dL)	93.8 ± 14.4	91.0 ± 13.2	0.235
HbA1c at enrollment (% and (mmol/mol))	5.32 ± 0.61 (35 ± 4.0)	5.27 ± 0.49 (34 ± 3.2)	0.589
Fructosamine at enrollment (mg/dL)	212.4 ± 27.8	209.7 ± 23.8	0.598
Triglyceridemia at enrollment (mg/dL)	203.9 ± 64.6	195.3 ± 76.6	0.502

Values are presented as mean ± SD for continuous variables and as *n* (%) for categorical variables. *p*-values correspond to between-group comparisons at baseline (ANOVA or chi-square test, as appropriate).

**Table 2:** OLIDIAG study: Primary outcomes.

	Control Diet GDM Group (*n* = 66)	Intervention Diet GDM-EVOO Group (*n* = 74)	Statistical Comparison
Insulin required at first visit (%)	9 (13.6)	13 (17.6)	1.288 (0.603–2.784) *p* = 0.524
Insulin required at pre-delivery (%)	36 (54.6)	31 (41.9)	0.758 (0.534–1.085) *p* = 0.135
Insulin required during the intervention (%)	27 (40.9)	18 (24.3)	0.595 (0.361–0.967) *p* = 0.036
Time to insulin initiation (weeks)	6.5	Median not reached	0.227 (0.074–0.698) *p* = 0.010 ^a^ 0.219 (0.070–0.679) *p* = 0.010 ^c^
Insulin dosage at term (units)	24.3 ± 22.6 ^a^ 23.3 ± 24.7 ^b^	26.8 ± 21.5 ^a^ 23.7 ± 24.9 ^b^	2.5 (−4.8–9.8) *p* = 0.645 ^a^ 0.4 (−11.6–12.4) *p* = 0.959 ^b^
Triglyceridemia (mg/dL)	257.6 ± 72.9 ^a^ 256.7 ± 82.5 ^b^	214.3 ± 73.6 ^a^ 213.7 ± 77.9 ^b^	−43.3 (−66.8–−19.8) *p* = 0.002 ^a^ −43.0 (−68.6–−17.4) *p* = 0.005 ^b^
Gestational weight gain (kg)	7.8 ± 5.7 ^a^ 8.1 ± 5.7 ^b^	7.6 ± 5.7 ^a^ 7.7 ± 5.4 ^b^	−0.2 (−2.1–1.7) *p* = 0.829 ^a^ −0.4 (−2.3–1.5) *p* = 0.646 ^b^

For categorical outcomes, values are presented as *n* (%); statistical comparison: relative risks (95% confidence intervals) and *p* values. For continuous outcomes, values are presented as mean ± SD; statistical comparison: mean differences (95% confidence intervals) and *p* values. For time-to-event outcomes (time to insulin initiation), values are presented as median time to event (when reached); statistical comparison: hazard ratio (95% confidence intervals) and *p* values. ^a^ Unadjusted estimates. ^b^ Adjusted values were obtained by ANCOVA including gestational age at delivery and pre-pregnancy BMI as covariates. ^c^ Hazard ratios were estimated by Cox proportional hazards regression adjusted for pre-pregnancy BMI.

**Table 3:** OLIDIAG study: Maternal outcomes.

	Control Diet GDM Group (*n* = 66)	Intervention Diet GDM-EVOO Group (*n* = 74)	Statistical Comparison
Term BMI (kg/m^2^)	33.6 ± 5.9 ^a^ 33.7 ± 6.1 ^b^	33.0 ± 6.1 ^a^ 33.1 ± 7.2 ^b^	−0.6 (−2.6–1.4) *p* = 0.555 ^a^ −0.6 (−2.9–1.7) *p* = 0.594 ^b^
Gestational age (weeks)	37.9 ± 1.2 ^a^ 37.9 ± 1.2 ^c^	38.3 ± 1.2 ^a^ 38.3 ± 1.2 ^c^	0.4 (0.0–0.8) *p* = 0.141 ^a^ 0.4 (0.0–0.8) *p* = 0.141 ^c^
Fasting glucose at term (mg/dL)	88.2 ± 11.7 ^a^ 88.2 ± 11.7 ^b^	88.68 ± 25.5 ^a^ 88.68 ± 25.5 ^b^	0.5 (−5.6–6.5) *p* = 0.898 ^a^ 0.5 (−5.6–6.5) *p* = 0.898 ^b^
Fructosamine at term (mg/dL)	196.8 ± 42 ^a^ 196.9 ± 86 ^b^	198.0 ± 45 ^a^ 198.3 ± 78 ^b^	1.2 (−13.2–15.6) *p* = 0.871 ^a^ 1.4 (−26.4–29.2) *p* = 0.923 ^b^
HbA1c at term (%)	5.53 ± 0.6 ^a^ 5.55 ± 0.5 ^b^	5.44 ± 0.5 ^a^ 5.45 ± 0.5 ^b^	−0.09 (−0.28–0.10) *p* = 0.322 ^a^ −0.10 (−0.27–0.07) *p* = 0.216 ^b^
TG/HDL cholesterol ratio at term	4.63 ± 1.7 ^a^ 4.67 ± 2.0 ^b^	3.70 ± 1.5 ^a^ 3.64 ± 1.8 ^b^	−0.93 (−1.46–−0.40) *p* = 0.001 ^a^ −1.03 (−1.65–−0.41) *p* = 0.002 ^b^
Excessive caloric intake (%)	6 (9.09)	6 (8.11)	0.892 (0.317–2.516) *p* = 0.836
Insufficient caloric intake (%)	11 (16.7)	9 (12.2)	0.729 (0.329–1.616) *p* = 0.447
Excessive carbohydrates intake (%)	16 (24.2)	11 (14.7)	0.612 (0.309–1.206) *p* = 0.160
Insufficient protein intake (%)	12 (18.2)	9 (12.2)	0.669 (0.306–1.455) *p* = 0.319
Excessive total fat intake (%)	6 (9.09)	5 (6.7)	0.743 (0.251–2.200) *p* = 0.608
Excessive saturated fat intake (%)	7 (10.6)	5 (6.7)	0.637 (0.222–1.817) *p* = 0.417
Excessive PUFAs intake (%)	2 (3.0)	3 (4.1)	1.338 (0.275–6.562) *p* = 0.745
Maternal complications composite ^d^ (%)	17 (25.8)	13 (17.6)	0.682 (0.363–1.280) *p* = 0.238
Hypertensive complications (%)	8 (12.1)	6 (8.1)	0.669 (0.253–1.757) *p* = 0.574
Placental complications (%)	2 (3.0)	2 (2.7)	0.892 (0.161–4.956) *p* = 0.907
Cholestasis (%)	3 (4.5)	3 (4.0)	0.892 (0.212–3.763) *p* = 0.886
Hemorrhages (%)	1 (1.5)	0 (0)	0 (0–3.390) *p* = 0.471
Preterm birth (%)	7 (10.6)	5 (6.8)	0.682 (0.363–1.280) *p* = 0.417
Total cesarean section (%)	44 (66.7)	43 (58.1)	0.872 (0.669–1.132) *p* = 0.383
Non-elective cesarean section (%)	28 (42.4)	24 (32.4)	0.764 (0.495–1.175) *p* = 0.222
Patients with labor (%)	34 (51.5)	41 (55.4)	1.076 (0.789–1.479) *p* = 0.646

For categorical outcomes, values are presented as *n* (%), statistical comparison: relative risks (95% confidence intervals) and *p* values. For continuous outcomes, values are presented as mean ± SD, statistical comparison: mean differences (95% confidence intervals) and *p* values. ^a^ Unadjusted estimates. ^b^ Adjusted values were obtained by ANCOVA including gestational age at delivery and pre-pregnancy BMI as covariates. ^c^ Adjusted values were obtained by ANCOVA including pre-pregnancy BMI as covariate. ^d^ Maternal composite: hypertensive complications, placental complications, cholestasis, hemorrhages, and preterm birth.

**Table 4:** OLIDIAG study: Neonatal outcomes.

	Control Diet GDM Group (*n* = 66)	Intervention Diet GDM-EVOO Group (*n* = 74)	Statistical Comparison
Large for gestational age (%)	5 (7.69)	5 (6.76)	0.878 (0.283–2.730) *p* = 0.831
Small for gestational age (%)	2 (3.08)	4 (5.41)	1.757 (0.389–8.039) *p* = 0.404
Neonatal weight (g)	3329 ± 466 ^a^ 3379 ± 442 ^b^	3227 ± 459 ^a^ 3232 ± 423 ^b^	−102 (−255–51) *p* = 0.194 ^a^ −147 (−291–−3) *p* = 0.047 ^b^
Neonates with complications (%) ^c^	17 (25.7)	14 (18.92)	0.730 (0.396–1.359) *p* = 0.331
Neonates with more than one complication ^c^ (%)	13 (19.7)	5 (6.76)	0.340 (0.133–0.870) *p* = 0.041
Hypoglycemia (%)	6 (9.2)	6 (8.1)	0.870 (0.312–2.470) *p* = 0.814
Hyperbilirubinemia (%)	7 (10.8)	9 (12.2)	1.130 (0.461–2.790) *p* = 0.797
Respiratory distress syndrome (%)	6 (9.2)	3 (4.05)	0.439 (0.124–1.539) *p* = 0.216
NICU requirement (%)	12 (18.2)	5 (6.8)	0.367 (0.140–0.939) *p* = 0.036
NICU requirement in the subpopulation with complications ^d^ (%) (*n* = 30)	12 (75.0)	5 (35.7)	0.476 (0.210–0.935) *p* = 0.030
NICU requirement in the subpopulation with respiratory distress syndrome (%) (*n* = 9)	6 (100)	2 (33)	0.667 (0.208–1.236) *p* = 0.333
NICU requirement in the subpopulation with hyperbilirubinemia (%) (*n* = 16)	4 (57.1)	5 (55.6)	0.972 (0.403–2.504) *p* = 0.949
NICU requirement in the subpopulation with hypoglycemia (%) (*n* = 12)	6 (100)	2 (33)	0.333 (0.097–0.760) *p* = 0.014

For categorical outcomes, values are presented as *n* (%); statistical comparison: relative risks (95% confidence intervals) and *p* values. For continuous outcomes, values are presented as mean ± SD; statistical comparison: mean differences (95% confidence intervals) and *p* values. ^a^ Unadjusted estimates. ^b^ Adjusted values were obtained using ANCOVA including gestational age at delivery and pre-pregnancy BMI as covariates. ^c^ Neonatal complications composite: hypoglycemia, hyperbilirubinemia, respiratory distress syndrome, shoulder dystocia, perinatal death, and NICU requirement. ^d^ Neonatal complications considered: hypoglycemia, hyperbilirubinemia and respiratory distress syndrome.

## Data Availability

The data presented in this study are available on request from the corresponding author due to privacy and ethical restrictions.

## Supplementary Materials

The following supporting information can be downloaded at: https://www.mdpi.com/article/10.3390/nu18071120/s1, CONSORT 2025 checklist.

## Registration

Registered in clinicaltrials.gov—Registration Number: NCT05120388 (15 November 2021).
